# Inclusion body myositis—health-related quality of life and care situation during phases of the “patien*ce* journey” in Germany: results from a qualitative study

**DOI:** 10.1186/s12955-023-02196-w

**Published:** 2023-10-10

**Authors:** Katja C. Senn, Simone Thiele, Laura Gumbert, Sabine Krause, Maggie C. Walter, Klaus H. Nagels

**Affiliations:** 1 University of Bayreuth, Chair of Healthcare Management and Health Services Research, Parsifalstrasse 25, 95445 Bayreuth, Germany; 2 Department of Neurology, Friedrich Baur Institute, LMU University Hospital, LMU Munich, Ziemssenstrasse 1, 80336 Munich, Germany; 3 SMA Europe, Im Moos 4, 79112 Freiburg, Germany

**Keywords:** Health-related quality of life, Inclusion body myositis, Social support, Qualitative research, Health services research

## Abstract

**Background:**

To understand the health-related quality of life (HRQoL) in inclusion body myositis (IBM) from a holistic perspective on the background of a complex care situation. The focus was on how the patient journey may be structured over the course of this rare disease.

**Methods:**

An exploratory qualitative study was performed via in-depth semi-structured interviews. Seven patients (males *n* = 5) with 2011 European Neuromuscular Centre (ENMC) IBM criteria from the German IBM patient registry were interviewed for this study. The dynamic network approach of resilience and the throughput-model of health services research were used to structure the qualitative analysis.

**Results:**

Our results suggest that IBM patients experience the holistic HRQoL and care situation typically in four phases: (1) uncertainty about physical vulnerability until diagnosis, (2) promising treatment approaches, (3) self-management and dyadic coping, (4) weak body, busy mind and caregiver burden. The homophonous in-vivo code “patien*ce* journey” describes the frequently reported emotional perspective of the patient journey. Although the overarching theme of perceived social support varied throughout these phases, a reliable patient-partner-dyad may lead to improved HRQoL in the long-term.

**Conclusions:**

New hypotheses for future quantitative research were generated to better understand the IBM patients’ burden in the long term. The identified relevance of social support emphasizes the patients’ need to handle IBM as manageable in medical settings. During exhausting phases of IBM progression, more effective care elements for patients and their partners could disclose varying needs. Strengthening multi-professional healthcare services via individualised informational, practical, or emotional support could improve HRQoL, especially since there is no curative treatment available so far.

**Supplementary Information:**

The online version contains supplementary material available at 10.1186/s12955-023-02196-w.

## Background

Inclusion body myositis (IBM) is the most prevalent inflammatory myopathy beyond the age of 50 with a two to threefold higher prevalence in males [1–3]. Asymmetric slow progression of proximal and distal muscle weakness as well as atrophy of quadriceps femoris, long finger flexors and tibialis anterior are typical clinical hallmarks of the disease [4–6]. Dysphagia is often underreported, although swallowing problems frequently occur during progression [4, 7]. IBM is in general not associated with a higher mortality, but severe complications of dysphagia, e.g., aspiration pneumonia, can contribute to premature death [1, 4, 8].

Individual approaches with intravenous immunoglobulin (IVIG) infusions, regular supportive rehabilitation and physiotherapy are recommended [9]. Treatment goals are maintaining physical function and strength, managing dysphagia and preventing complications and side effects [6, 9, 10]. Unfortunately, there is no curative treatment yet available [6, 9, 11].

The chronic and disabling disease impacts health-related quality of life (HRQoL) multidimensionally [12–14]. HRQoL is either assessed with qualitative methods or with generic patient-reported outcome measures (PROMs), specific neuromuscular or disease-specific PROMs [15–18]. Recent IBM-specific PROMs represent only physical, and partly social dimensions of HRQoL, although it is evident that psychological outcomes mediate or moderate HRQoL in neuromuscular diseases (NMD) and old age [13, 19–21]. Natural history studies or long-term follow-ups measure IBM progression merely with physical outcomes [1, 14, 22].

Previous research stipulates assessing HRQoL and the complex care needs in IBM patients’ everyday lives from a comprehensive perspective [15, 23–25]. Most prior research has neglected to provide such sufficient insights into the specific HRQoL in IBM. So far, key problems are inconsistent diagnostic criteria or the lack of direct patients’ perspectives within the actual ecosystem of care [13, 25–27]. A deeper understanding of HRQoL will improve clinical practice, value co-creation and economic assessments of future therapies for IBM [28–30].

As first part of a sequential mixed-methods series, this exploratory qualitative study aims at understanding HRQoL in IBM patients from an in-depth holistic perspective on the background of a complex care situation. With the term “holistic” we want to emphasize the need for a comprehensive understanding of all relevant HRQoL dimensions in IBM (physical, psychological and social) and possible multifactorial determinants in this complex system of patients’ everyday life. We focused on structuring the patient journey from a patients’ view, on patient reported categories of holistic HRQoL and complex care situations in the German healthcare setting.

## Methods

A qualitative ethnographic methodology [31] was applied to gain exploratory insights of HRQoL in IBM patients. The interview guide was based on a systematic review according to physical, psychological and social dimensions of HRQoL in IBM [32]. After peer feedback and a pre-test, the interview questions were phrased more openly.

IBM patients identified via the 2011 European Neuromuscular Centre (ENMC) diagnostic criteria "clinicopathologically defined" or "clinically defined" or "probable" IBM [26, 27] were recruited from the German IBM patient registry (www.IBM-registry.org) via telephone or e-mail by the registry curator at the Friedrich-Baur-Institute in Munich. A written information letter with study details and information about the research collaboration was sent out prior to participation. Patients were eligible to participate if they were ≥ 18 years old, able to give informed consent and German-speaking. Patients with acute suicidality, psychiatric personality disorders or less than one year of remaining life expectancy due to severe comorbidities were excluded. Written informed consent was obtained from all participants. No personal relationship between patients and researchers pre-existed.

The study followed ethical guidelines, with respect to the Declaration of Helsinki. Ethical approval was obtained from the ethics committee at the University of Bayreuth and at the Ludwig-Maximilians-University Munich.

The material was supplemented with patients’ sociodemographic characteristics, memos and ethnographic fieldnotes [31]. The interviews were anonymized via protocol [33]. Audio recordings of the interviews were transcribed verbatim [34] with f4transcipt software (version 7, dr. dresing & pehl GmbH, Marburg, Germany).

Theoretical saturation was reached after seven interviews (male *n* = 5), having no dropouts or refusers. In accordance with the saturation principle [35], a maximum contrast of cases was pursued in terms of age, disease duration and use of mobility devices.

The three-step qualitative content analysis [31] was performed with the software f4analyse (version 3.0.0., dr. dresing & pehl GmbH, Marburg, Germany). Firstly, an open coding was applied inductively with paper–pencil. Secondly, the basis for the inductive-deductive selected coding were the formerly identified HRQoL dimensions of the literature search. Abduction was observed after the selected coding, meaning that parts of the material and phenomena could not been assigned to the selected codes. An epistemological solution was developed in order to clarify the relationship between the factors influencing HRQoL and its dimensions. The qualitative analysis according to Emerson [31] is a reflexive, dialectic process aiming to explore inductively new concepts and perspectives. Therefore, integrative memos encouraged to search for existing theories and models to better capture the changing patients’ perceptions. Lastly, the framework of the dynamic network approach of resilience [36] and the throughput-model of health services research [37] structured the focused coding. The appraisal process of IBM patients’ HRQoL seemed to resemble with the understanding of resilience as a dynamic network [36]. Moreover, the throughput-model [37] represents the health care system in order to illustrate the input (influencing factors of health care), the throughput (structures and processes of health care), the output and outcome. Thus, the selected coding was substituted with the focused coding to represent the context of the phenomenon (reflexivity) within the different contacts of health care services along the patient journey (applied concept: [37]) and the dynamic and interconnected dimensions of HRQoL (applied concept: [36]). Through this proactive application of processuality, reflexivity and adequacy it was possible to explain the relevance and importance of the flexible and adaptive HRQoL perceptions in IBM in a model-like manner.

The codes and codebook were reviewed for comprehensibility. To ensure triangulation interim findings were discussed interprofessional [38]. The consolidated criteria for reporting qualitative research (COREQ) checklist [39] guided this study and report.

## Results

Seven patients with diagnosed IBM were interviewed between July and November 2020. All patients were retired, married and lived together with their spouse. More characteristics shows Table 1.

**Table 1:** Patients' sociodemographic and disease characteristics

**Characteristic**	**Total** **(*n* = 7)** [Min–Max; Mean]	**Women** **(*n* = 2)** [Min–Max; Mean]	**Men** **(*n* = 5)** [Min–Max; Mean]
Age (years)	66–81; 76.7	80; 80	66–81; 75.4
Duration since diagnosis (years)	2–17; 9.4	4–6; 5	2–17; 11.2
Age at symptom onset (years)	56–73; 62	62–73; 67.5	56–63; 59.8
Physical functioning (sIFA)	44–101; 87	89–94; 91.5	44–101; 85.6
**ENMC diagnostic criteria**	absolute; %	absolute	
Clinicopathologically defined IBM	4; 57%		4
Clinically defined IBM	2; 29%	2	
Probable IBM	1; 14%		1
**Mobility devices**	absolute; %	absolute	
Canes to some extent	1; 14%		1
Wheelchair to some extent (manual wheelchair)	3; 43%	2	1
Completely wheelchair users (power wheelchair)	3; 43%		3
**Severe patient relevant comorbidities prior to diagnosis**		absolute	
Cervical damage due to motorcycle accident			1
Gunshot wound to hand and foot			1
Lesions of peroneal nerve due to an accident			1
Cardiovascular disease			1
Bronchial asthma		1	
Implantation of total knee endoprosthesis		1	
**Former socio-professional category**	absolute; %	absolute	
Workers/employee	3; 43%	2	1
Senior executive/higher intellectual professions	4; 57%		4
**Type of health insurance**	absolute; %	absolute	
Statutory	2; 29%	1	1
Private	5; 71%	1	4

*ENMC* European Neuromuscular Centre [26, 27]; The Sporadic Inclusion Body Myositis Physical Functioning Assessment (sIFA) [40, 41] is a PROM with a 0–10 (0 = no difficulty, 10 = unable to do) numerical rating scale with 11 items covering lower and upper body functioning and general functioning

Six interviews were conducted face-to-face, five at the patients’ home, one at the Friedrich-Baur-Institute; one via telephone, due to social restrictions of the COVID-19 pandemic.

Two patients wanted their spouse to be present all the time or part-time (*n* = 4), respectively the grandson (*n* = 1). Two patients were interviewed on their own. A few field notes were sent to one patient to clarify ambiguities. The interviews lasted on average 1.30 h (min 0.54 h; max 2.06 h). Altogether, 10.34 h of audio material was collected.

### Phases of HRQoL and care situation: perceived social support as overarching theme

**Fig. 1:**
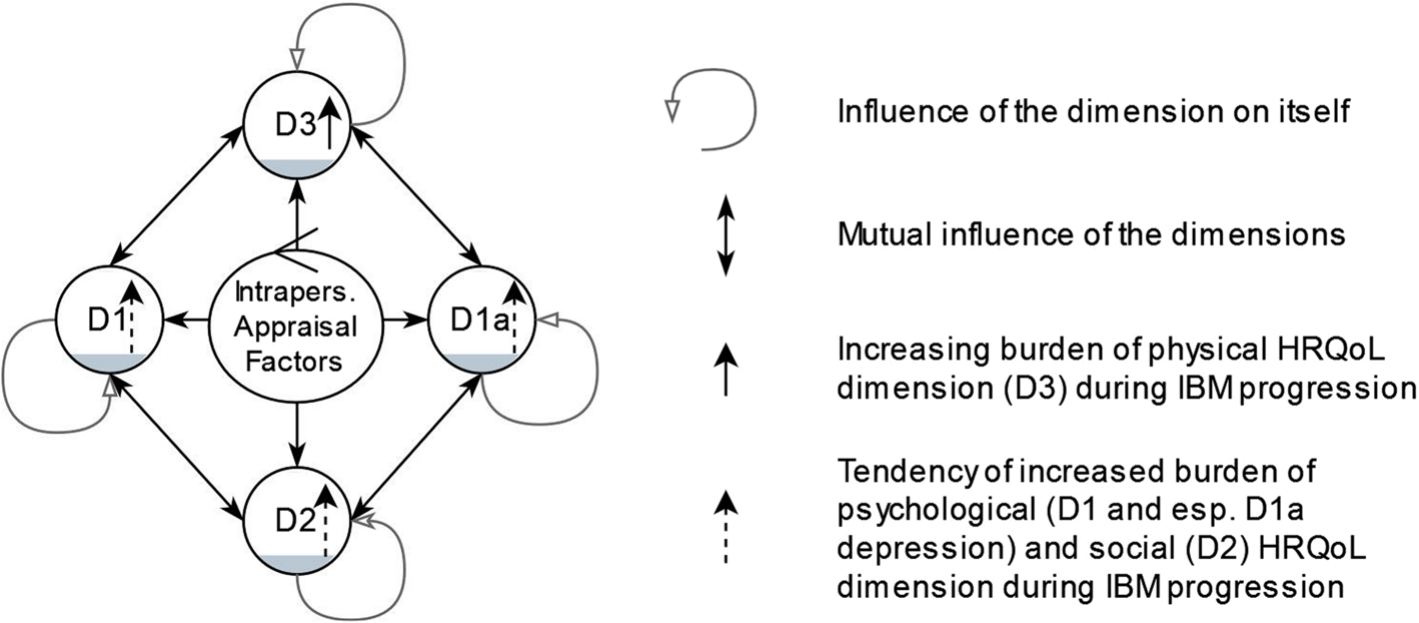
Dimensions of HRQoL in IBM and suggested tendencies due to physical progression. Applied and adapted dynamic intrapersonal appraisal of HRQoL in IBM patients during progression, based on theoretical considerations of symptom network appraisal [35]. The codes of HRQoL appraisal are shown as bowls for psychological (D1), depressive (D1a), social (D2) and physical (D3) dimension. Intrapersonal appraisal factors summarise the codes: coping (emotional and problem-oriented), coherence, self-efficacy expectation, religiousness and spirituality. We explicitly used this coding and presentation to demonstrate the indicated time-dependent connections of the HRQoL dimensions (two sided arrows) found in the interview material. The grey filling of the bowls illustrates the extent of impairment of HRQoL dimensions at exemplary time point during the four phases

All patients reported dynamic interdependences of physical, psychological, and social HRQoL during IBM progression (Fig. 1). Time-varying, but temporal constant, perceptions of the patients were described as phases. Thus, the results suggest four phases to structure the HRQoL and care situation during the IBM patient journey: (1) uncertainty about physical vulnerability until diagnosis, (2) promising treatment approaches, (3) self-management and dyadic coping and (4) weak body, busy mind and caregiver burden. Therefore, the *in-vivo* code and homophony “patien*ce* journey” explicates the reported necessary emotional patience to persevere slowly progressive or stable phases, as well as exacerbations with sudden deteriorations of HRQoL.

**Fig. 2:**
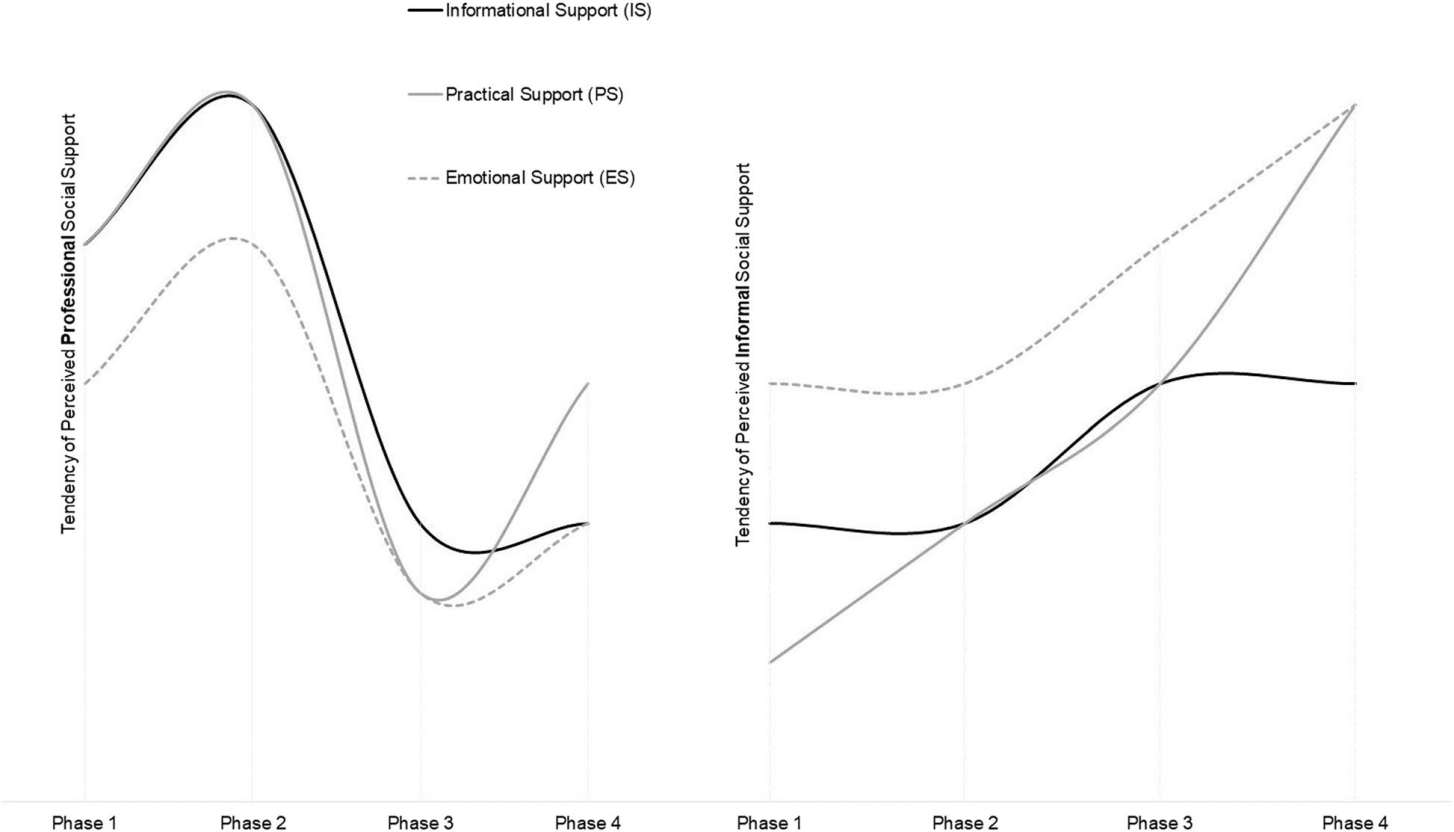
Tendency of perceived support during the four phases. Due to the qualitative design of our study, the graphs should depict the suggested hypotheses for the tendencies of IBM patients’ perceived support in the German healthcare system during the four phases

Moreover, our identified overarching theme in these phases is the perceived social support, specified as informational, practical, and emotional support (IS, PS, ES) (Table 2). Social support was defined comprehensively to encompass not only informal, but also professional input [42]. The phases of physical, psychological and social HRQoL and care situation seem to have an interindividual timing and to be impacted by perceived social support. Figure 2 illustrates the interpreted tendencies of perceived support during the IBM patient journey. The applied theoretical conceptualisation and coding framework is shown in Additional file 1.

**Table 2:**
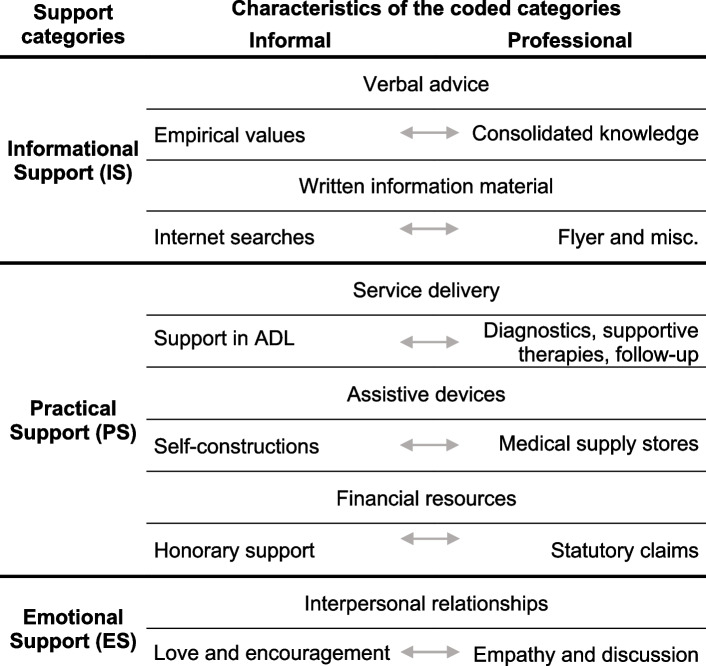
Main characteristics of the coded support categories

*ADL* Activities of daily living; Support categories according to Schwarzer [42]

### Phase 1: Uncertainty about physical vulnerability until diagnosis

Overall, patients initially reported an increased physical vulnerability with an intangible onset. Compared to the almost stable social HRQoL, the most common psychological response was feeling uncertainty. The first phase seems to last several months or even years.

Physical behaviour adjustments, changed motion sequences while deterioration of walking ability and intermittent falls were eventually interpreted as pathological: “It got worse and worse with the falls, more often. It was just that the feet were lagging” (P06 (patient interview code), paragraph (para.) 5). Decreased physical HRQoL was minor underpinned with less grip strength or swallowing problems as initial symptoms.

The social HRQoL seems not to be limited in this phase. No one reported constraints in the activities of daily living (ADL) or employment status.

Psychologically, the inexplicable vulnerability and laboratory findings were perceived as uncertainty. It appeared, that patients developed a sense of hazard cognition to manage resources or prevent falls: “I said: "I'm also thinking about the way back. Let's not go too far."” (P05, para. 53). Continuous informal ES mostly promoted patience to persevere this first phase of the total IBM “patien*ce* journey” and actively seek professional support until the patients receive a final diagnosis. Interestingly, a confirmed diagnosis of IBM by neuromuscular specialists (NMS) was vital for patients to satisfy an informational need. The negative emotional coping turned into more positive with time: “It pulls your feet away; And then there's just one thing: you have a wife, you have a family, and you just have to look ahead” (P06, para. 265). At the time of diagnosis, empathetical and honest professional ES was appreciated just as IS in the form of individual recommendations. The evidence that IBM does not lead to premature death promoted optimism in most patients.

### Phase 2: Promising treatment approaches

A prominent theme in the second phase was to pinpoint various treatment approaches. Patients described slow but steady physical deterioration and social constraints. Positive coping abilities and emotional responses were minor underpinned by professional support. The data also indicated a varying duration of phase 2, mainly depending on how long patients perceive professional support as most helpful.

More specifically, professional PS (e.g., physical-, occupational therapy) and independently performed exercises by patients seem to enhance the physical HRQoL. Most patients aimed at muscle training, while they felt slightly impaired functioning. Some patients reported side effects of IVIG (thrombosis, hypertension, paravasat, dermatologic problems) and “warn [ed] about being too careless with immunoglobulins” (P01, para. 89). Hair loss was mentioned once, while participating in a pharmacological trial. For some, naturopathy mitigated pain or promoted muscular strength. Improvements or stabilisation of physical HRQoL due to pharmacotherapy was rarely reported; placebo effects were suspected twice. Most patients still walked independently, used crutches or canes occasionally. Walking and carrying objects simultaneously were challenging. Diminutive adaptions appeared important: “Whether you hold on to the banister just for safety or for fear is also important […] or […] to get upstairs at all […]. That's quite a big difference” (P02, para. 891–893). Swallowing problems were characterized as being less communicative during dinner or as experiencing embarrassment in public while choking.

Problem-oriented coping was common as “doing well with close relatives, with friends, with travel” (P04, para. 210). It appeared beneficial, “to have the time” (P02, para. 755) and “be relaxed” (P02, para. 758) at home and to afford mostly self-paid adjustments (e.g., banisters, higher toilets). Behaviour adjustments (e.g., changing hobbies) and commodity items (e.g., shoulder bags) served as assistive devices (AD), enabling social independency and flexibility.

AD were dominantly assessed positively as self-determination and security (psychological). Thereby, professional IS and ES “saved the mood” (P03, para. 595) and promoted acceptance and accurately customisation of AD. In contrast, anxiety, hopelessness or impaired self-efficacy expectations arose with minor empathetic and need-oriented professional IS and ES: “She [logopaedic] first explained to me all the things that happen to me with this dysphagia. She ended with a feeding tube. […] This frightened me a lot” (P04, para. 112–115). Information on available healthcare services provided security, whereas the cost coverage of IVIG distressed. Most perceived ES and IS from self-help groups as too general or scaring “steppage gait, polyneuropathy, […] ALS and all that shit” (P06, para. 145). Downward comparisons as “it is this [IBM] disease” and not “Pompe or other possibilities” (P02, para. 447) were mentioned. Anger, frustration, or shame were reported as thinking about visible vulnerability, physical ageing and barriers in the environment.

### Phase 3: Self-management and dyadic coping

The transition from phase 2 to phase 3 could be characterized by intensified physical and social burden and the perceived run out of professional support as patients and caregiver predominantly cope dyadic. Psychological deteriorations appeared to be more prominent.

Precisely, the progression was more experienced as subphases, where “the disease strikes again and again” (P04, para. 139). Exacerbations were tremendous, relating to physical and psychological HRQoL: “I felt good, strong and then I get this hit of fracture and I don't even know how to get out of it” (P07, para. 16), or to social HRQoL: “I can't drive the boat through the harbour anymore, [so] I can't drive it at all.” (P03, para. 142).

The decline of strength, functioning, digestive disorders and chill was perceived as impaired physical HRQoL. Immobilisation and the need to rest happened faster than expected. The appropriate utilisation of AD was challenging. Many “always exaggerate it until” they “dropped” (P03, para. 110) painfully. More acute PS after falls was needed. Patients with severe dysphagia avoided certain foods and increased fluid intake. With professional PS (esp. physiotherapy), the patients now aimed more at maintaining mobility and functioning. Effects of IVIG > 6 months (*n* = 2) fluctuated between “no longer measurable” (P01, para. 28) or improvement. “Occasional examinations” at follow-ups with NMS were mainly perceived as “useless” (P01, para. 192). From patients’ views, clinical appointments documented physical deterioration, influenced work and leisure time but for some also promoted hope.

Rising PS was noticed for personal hygiene, domestic care or social participation. Self-paid services and multifunctional AD beyond statutory claims indicated a pleasing social HRQoL. Without appropriate information, “adventurous” (P03, para. 165) AD were customised to maintain mobility or lower the fear of falls. Most “got used to the visible disability” (P05, para. 75) and spend their time at home: “[Friends] always arrive with their cake, so that we are here in my familiar surroundings, where I have everything” (P05, para. 250). Loneliness was rarely reported.

The psychological burden was highlighted, especially symptoms of depression: “At least half of the strength that is lost goes out of the body, but also out of the mind. […] Sometimes even much more from the mind than from the body” (P01, para. 363–365).

The need of IS, how the progression “concretely affects the ability to move […] in the end” (P05, para. 216), was common. Patients were disappointed if they noticed physical exacerbations: “I thought it could not progress any further” (P07, para. 5). Lower perceived professional support after phase 2 and thus more self-management was mainly coped with negative emotions and distrust in healthcare. Two patients refused follow-ups with NMS, while others valued the “exchange of experience” (P01, para. 15). Patients felt distress by the rising costs of AD and the justification of their necessity to health insurances. Therefore, the support by physicians or therapists was mainly perceived as helpful, especially after tremendous exacerbations “when professionals say: "Go there, you're entitled to this, you need that"” (P06, para. 45). Hidden support let thereby patients “forget again that something is not possible” (P07, para. 133) indicating self-efficacy and positive coping. Self-help groups, ongoing treatment and religion served solely for some patients as most helpful coping strategy. But PS and vast ES from spouses were frequently a foundation for high HRQoL. The important issue of dyadic coping in the long-term was consistent in all interviews.

### Phase 4: Weak body, busy mind and caregiver burden

Overall, IBM was in the fourth phase experienced as being “enclosed” in the body. Patients aimed at staying mentally healthy and active. The patients’ attitude towards support was now more to decrease the primary caregivers’ burden and postponing their own needs.

In detail, physically “the worst thing [was] this complete inability to move” (P03, para. 102) and the reliance on AD like electric wheelchairs. Inadequate positioning caused pain (e.g., joint pain). Swallowing problems increased, but no one needed special nutrition or tube feeding. Ageing seems to intensify physical vulnerability. Especially during the treatment of comorbidities, patients perceived professional PS as inappropriate or too little, mentioning “pressure sore on the back” (P03, para. 759) or too much “feeding” (P04, para. 67).

The social “circle [got] smaller and smaller” (P03, para. 208). Some features of AD like “toilet showers, which also have a blow dryer ((laughs))” (P04, para. 300) created intimate personal hygiene more pleasant for both caregiver and patient. IS was needed to arrange home care. “Care work is not really the right thing for an 83-year-old [wife]” (P03, para. 447) but nursing homes were predominantly “no alternatives” (P03, para. 793), as expecting lower HRQoL. Professional PS was valued if it relieved the rising caregiver burden, “to make it as comfortable as possible for them [caregiver]” (P043, para. 65).

A key theme was the gratitude being mentally healthy, like “not suffering from dementia” (P06, para. 61). Negative emotions appeared mainly from inappropriate professional support concerning the common close patient-partner-dyad: “365 days, 24 h, no old sow works for 728 euros. But the relatives.” (P06, para. 344).

## Discussion

For the first time, four phases how IBM patients in Germany experience the HRQoL and care situation from an in-depth view were suggested. We aimed at structuring typical and different holistic HRQoL categories with exploratory qualitative methods.

Overall, physical progression was accompanied with continuous social and psychological HRQoL changes that shape the IBM patient journey also to an emotional “patien*ce* journey”. To achieve high HRQoL, patients favoured timely informational, practical, and emotional support. Our applied coding framework [36] was justified by these highly mentioned psychological dimensions, being consistent with other findings in IBM and NMD [13, 16].

In addition to physical milestones in IBM or somatic disorders [14, 19, 43], our outlined four phases try to represent the patients’ perceived holistic interplay of HRQoL and social support during IBM progression. After diagnosis and treatment approaches (phase 1–2), the input of perceived support changed from professional to informal. Accordingly, caregiver burden and dyadic-coping affected patients’ HRQoL increasingly as of phase 3. Incremental professional PS in phase 4 was highly relevant to relief the caregivers. The interindividual timing of these four phases suppose the variability in IBM [14, 25].

### Clinical implications

The HRQoL of IBM patients could be improved if the need of professional IS and ES in the phases 3 and 4 is covered by the healthcare system. The disease burden from IBM patients and their caregivers could be relieved as the manageable aspects of IBM (e.g., dyadic-coping strategies) are fostered in clinical practice [44, 45].

The results emphasise the need to understand how social support impacts HRQoL in chronic neuromuscular diseases [46, 47]. Our study underlines heterogeneous needs of IS, ES and PS during the patient journey as physical, psychological and social exacerbations increased as of phase 3 [25]. Additionally, the results suggest that psychosocial interventions might be implemented as an integral part in the follow-ups with NMS in medical settings to enhance patients’ health literacy, coping, self-management and AD management. Therefore, guidelines of more prevalent diseases can serve as a best practice example, although even there are evidence-practice-gaps in Germany [48, 49].

Due to scarce evidence of psychological interrelations in NMD and the role of social support in resilience outcomes, specific and sensitive psychological PROMs like the Hospital Anxiety and Depression scale (HADS) [50] combined with IBM-specific physical PROMs (IBM Functional Rating Scale, IBM-FRS [51]; Sporadic Inclusion Body Myositis Physical Functioning Assessment, sIFA [41]) and HRQoL assessments could enrich the clinical knowledge about the progression from a holistic view [36, 46]. Such PROMs could be implemented in existing digital health applications for NMD [52] and be customised for the monitoring of IBM patients. Therefore, patient relevant deteriorations might be indicated early, especially after sudden exacerbations, and initiate timely professional support in addition to routine follow-ups.

More importantly, prioritising the prevention and diagnosis of depression ab initio in the clinical management of IBM patients could avoid possible cascade effects in elderly, frail people with a low socioeconomic status. They are at higher risk for decreased healthcare utilisation, self-management and negative coping, which correlates negatively with informal cooperativeness and hence entails minor social support and HRQoL [53–57].

The results might include normal aspects of ageing, but IBM patients seem to pass through the gerontological “third phase of life” faster and might undergo a premature high age (fourth phase of life) [58]. As of phase 3 of our IBM “patien*ce* journey”, the cognitive, affective and social vulnerabilities as well as individualisation- and transformation tendencies were frequently observed as increasing in high age [59]. Nevertheless, response shift could be a reason for the positive appraisals in our sample [60].

### Research implications

As bureaucratic obstacles and high private payments could point towards an economic burden for patients and their families, this was sparsely reported in our sample [14]. One reason for this finding could be the high socioeconomic status of our patient group. According to other studies [61, 62], our findings warrant cost-of-illness studies to identify the societal burden of IBM [63]. Future economic evaluations of pharmacological and psychosocial treatments might benefit from respective studies to allocate all inputs of care effectively, especially for the IBM patients-partner-dyad [63]. Our four phases generated new hypotheses for future quantitative research and contributes to understand the “inner circle” of IBM patients’ burden, as designated from the International Myositis Assessment Clinical Study Group (IMACS) [23].

### Strengths and limitations

Within the recently implemented patient registry, it was hardly possible to identify singles with low socioeconomic status, severe dysphagia or ones who are bedridden or domiciled in nursing homes. This could justify a selection bias. Health inequalities might explain this underrepresentation of patients, who tend to utilise less healthcare services [64]. A strength of our study was the balanced IBM sample referring to age, sex, age of IBM onset, disease duration, health insurance types and former occupation. Additionally, explicit diagnostic criteria and measurement of functional status yield transparent interpretations. The presence of some spouses in the interviews might be minor a social desirability bias, rather pointing out the patient-partner-dyad in our trustful interviews, where even intimate themes were reported, other than in comparable studies [65, 66].

## Conclusion

To sum up, our findings suggest for the first time four patient relevant phases of HRQoL during IBM progression. The results emphasise further in-depth and quantitative investigations on the care situation and HRQoL in IBM patients. Multi-professional healthcare services could improve the patients’ HRQoL during the tedious and exhausting phases of progression; only a thorough understanding of the patient journey enables an appropriate reaction to the patients’ and caregivers’ changing physical, psychological and social needs.

### Supplementary Information

**Additional file 1. ** Coding frame. Representation of reported HRQoL and care situation of an IBM patient with the multiple categories of the applied network coding frame at a several time point.

## Data Availability

Data supporting the conclusions in this article are included within the article itself. The datasets used during the current study are available from the corresponding author on reasonable request.

## Abbreviations

AD: Assistive devices
ADL: Activities of daily living
COREQ: Consolidated Criteria for Reporting Qualitative Research
ENMC: European Neuromuscular Centre
ES: Emotional support
HRQoL: Health-related quality of life
IBM: Inclusion body myositis
IBM-FRS: IBM Functional Rating Scale
IMACS: International Myositis Assessment Clinical Study Group
IS: Informational support
IVIG: Intravenous immunoglobulin
NMD: Neuromuscular diseases
NMS: Neuromuscular specialists
PS: Practical support
PROMs: Patient reported outcome measure
sIFA: Sporadic Inclusion Body Myositis Physical Functioning Assessment
