# Supplementary material for: Inclusion body myositis—health-related quality of life and care situation during phases of the “patience journey” in Germany: results from a qualitative study
Source: Health Qual Life Outcomes. 2023 Oct 10;21:111. doi: 10.1186/s12955-023-02196-w (PMC10566017; doi:10.1186/s12955-023-02196-w)
Supplement: Supplementary file 1 — Additional file 1. Coding frame. Representation of reported HRQoL and care situation of an IBM patient with the multiple categories of the applied network coding frame at a several time point. [file 12955_2023_2196_MOESM1_ESM.pdf]

Additional file 1. Coding frame. Representation of reported HRQoL and care situation of an IBM patient with the multiple categories of the applied network coding frame at a several time point.

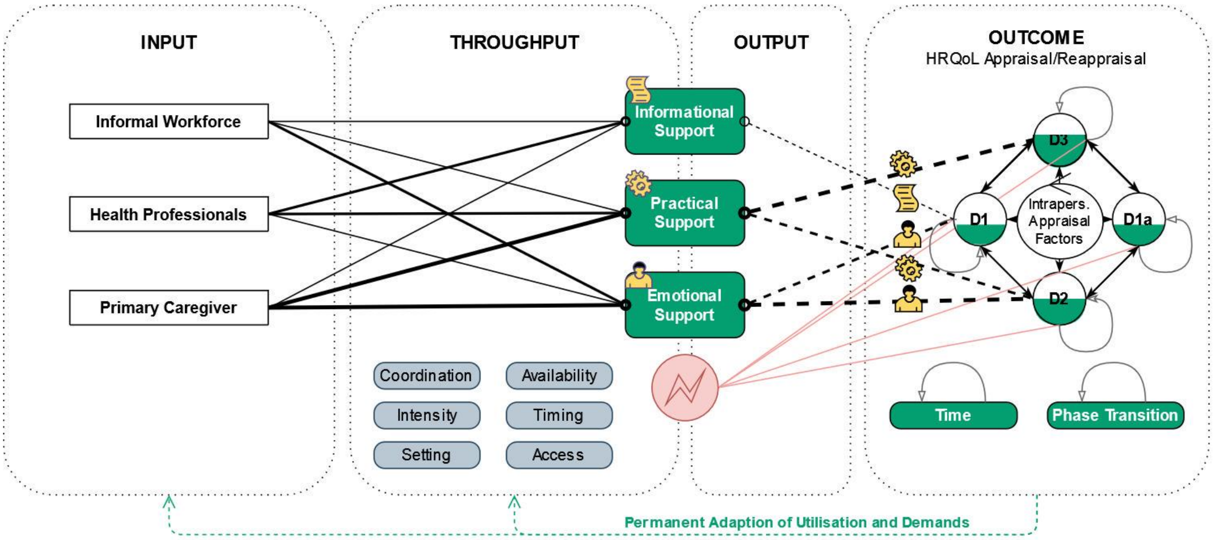

The illustrated HRQoL appraisal (outcome) was adapted and modified with the dynamic resilience framework.[1] The throughput-model of health services research [2] was applied as a framework for coding the complex care situation and in-depth structured with the perceived social support as informational, practical and emotional support [3]. The thickness of the left lines, connecting input and the different support options, represents the extent of perceived support at an exemplary time point of a patient's "patience journey". The thickness of the dashed lines, connecting the support options with the dimensions of HRQoL appraisal, represents the importance of the respective support output for HRQoL appraisal. The red thunderbolt symbolizes how exacerbations might therefore impact the HRQoL dimensions. The grey boxes in the throughput represent the patient relevant aspects of individualised health care

services. The green boxes in the outcome represent the dependence of varying HRQoL and support appraisal in the four phases.

References

1. Kalisch R, Cramer AOJ, Binder H, et al. Deconstructing and Reconstructing Resilience: A Dynamic Network Approach. Perspect Psychol Sci. 2019;14:765–77. doi:10.1177/1745691619855637.
2. Schrappe M, Pfaff H. Einführung in die Versorgungsforschung. In: Pfaff H ed. Lehrbuch Versorgungsforschung: Systematik - Methodik – Anwendung. 2nd ed. Stuttgart: Schattauer; 2017;1–68.
3. Schwarzer R. Psychologie des Gesundheitsverhaltens: Einführung in die Gesundheitspsychologie. 3rd ed. Göttingen: Hogrefe; 2004.
